# Neural shape completion for personalized Maxillofacial surgery

**DOI:** 10.1038/s41598-024-68084-5

**Published:** 2024-08-27

**Authors:** Stefano Mazzocchetti, Riccardo Spezialetti, Mirko Bevini, Giovanni Badiali, Giuseppe Lisanti, Samuele Salti, Luigi Di Stefano

**Affiliations:** 1 eDIMES Lab – Laboratory of Bioengineering, Department of Medical and Surgical Sciences, University of Bologna, Bologna, Italy; 2 Department of Computer Science and Engineering (DISI), University of Bologna, Bologna, Italy; 3 Oral and Maxillo-Facial Surgery Unit, IRCCS Azienda Ospedaliero-Universitaria di Bologna, Bologna, Italy; 4 Department of Biomedical and Neuromotoric Science (DIBINEM), University of Bologna, Bologna, Italy

**Keywords:** Shape completion, 3D deep learning, Maxillofacial surgery, Surgery planning, Personalized medicine, Engineering, Medical imaging

## Abstract

In this paper, we investigate the effectiveness of shape completion neural networks as clinical aids in maxillofacial surgery planning. We present a pipeline to apply shape completion networks to automatically reconstruct complete eumorphic 3D meshes starting from a partial input mesh, easily obtained from CT data routinely acquired for surgery planning. Most of the existing works introduced solutions to aid the design of implants for cranioplasty, i.e. all the defects are located in the neurocranium. In this work, we focus on reconstructing defects localized on both neurocranium and splanchnocranium. To this end, we introduce a new dataset, specifically designed for this task, derived from publicly available CT scans and subjected to a comprehensive pre-processing procedure. All the scans in the dataset have been manually cleaned and aligned to a common reference system. In addition, we devised a pre-processing stage to automatically extract point clouds from the scans and enrich them with virtual defects. We experimentally compare several state-of-the-art point cloud completion networks and identify the two most promising models. Finally, expert surgeons evaluated the best-performing network on a clinical case. Our results show how casting the creation of personalized implants as a problem of shape completion is a promising approach for automatizing this complex task.

## Introduction

In maxillofacial surgery, the surgical specialty dealing with the treatment of diseases of the head and neck region, a common occurrence is the need to treat malformations of the craniofacial skeleton, whether congenital, developed during growth, caused by trauma or surgical resection. The use of image-guided surgery, virtual surgical planning in CAD software and the production of 3D printed anatomical models and patient-specific implants are nowadays common practices, improving post-operative results and reducing surgical times ^1′2^. The current approach used in surgical planning is the manual manipulation of 3D medical imaging data (e.g. meshes obtained from CT scans) to simulate the correction of the malformation targeted by surgery. This approach is time-consuming, requires dedicated technicians and clinicians, and often requires sculptor-like skills that are largely operator-dependent.

In the field of computer vision, the task of automatically processing partial 3D shapes to coherently complete them, known as *shape completion*, has received a lot of attention in recent years ^3^. This task bears a resemblance to the problem faced by clinicians when performing surgical planning. However, shape completion algorithms are typically trained and tested on ShapeNet ^4^, a dataset of point clouds depicting common objects such as chairs and tables. The geometry of these objects is significantly different from the craniofacial skeleton and the level of detail is also orders of magnitude smaller: ShapeNet point clouds usually have 2048 points, while meshes obtained from CT scans usually have hundreds of thousands of vertices. In addition, shape completion algorithms generate missing parts by simulating the acquisition of the shape by a 2.5D sensor, with missing parts spread across the object surface due to self-occlusions. In contrast, malformations in the craniofacial skeleton typically involve only one contiguous area of the skull, which is completely missing.

Despite these differences, the impressive performance of state-of-the-art shape completion models is promising. If the same level of completion quality could be achieved for craniofacial meshes, the reconstructions could be used as starting points in surgical planning to greatly reduce planning time and provide patient-specific hints to clinicians. Therefore, in this work, we explore the feasibility of using modern neural networks designed for shape completion to tackle the problem of automatic craniofacial skeletal reconstruction.

The contributions of our work can be summarized as follows:

- by using CQ500 ^5^, we create a dataset to study point cloud completion for craniofacial skeletal reconstruction. Meshes in the dataset have been manually cleaned and aligned so as to be able to automatically extract the point clouds on which virtual defects are applied. We will make it publicly available to encourage further research on this topic.
- we evaluate several state-of-the-art neural network architectures for point cloud completion on the problem of craniofacial skeletal reconstruction;
- we outline a whole operational pipeline that would allow a clinician to highlight the defect region in the input mesh and obtain a high-resolution, high-quality proposal of a patient-specific completed skull.
- differently from existing techniques^6–8^ that focus on the reconstruction of defects localized in the neurocranium, which usually have a smooth surface, we also take into account the splanchnocranium, which exhibits a more complex structure, and report separate performance for this challenging region.

## Related work

Thanks to the ability to generate 3D models from patient data, more medical applications in custom prosthetics and implants are nowadays designed following a digital reconstruction approach.

Works in this area can be grouped into two macro-areas: methods based on computer-aided design and data-driven approaches (i.e., deep learning methods). Depending on the reconstruction strategy, the former can also be divided in: mirroring, surface interpolation, deformed template and slice-based reconstruction. Mirroring-based methods aim at exploiting the skull symmetry to retrieve the missing geometry^2,9^. In order to reconstruct the defective region, this approach proposes to reflect the non-defective side of the skull. Even if this solution achieves good results, it requires performing several manual operations, such as the computation of the symmetry plane. Moreover, it can be applied only on unilateral skull damages: indeed, if the defect crosses the sagittal plane the symmetry property no longer holds. Methods based on surface interpolation generate an approximation of the defective region to complete the skull shape^10,11^. These methods allow for adjusting the resulting fit by tuning the parameters of the interpolation. The advantage of these approaches is the continuity at the boundary of the defective region. However, they lack constraints for the reconstruction of the internal part of the defect. For these reasons, they work well with small defects but they are not able to reconstruct large defects properly. Template deformation starts from a database of 3D skull models to generate a reference shape (i.e., a template), exploiting some statistical tools. Other solutions^12–14^ use this reference model combined with geometric morphometrics to obtain reconstruction. On one hand, this approach works well for reconstruction of large-scale defects and is able to deal with bilateral defects. On the other hand, the results are strongly influenced by the quality of the template and its similarity with the target patient’s anatomy. Extensive and time-consuming CAD manipulation is also necessary in nearly all cases. Slice-based reconstruction aims at fitting a mathematical curve on the bone contour by minimizing the energy of a functional. The curve in each CT image is modeled starting from an oval shape. In Chen et al.^10^, the authors apply the Active Contour Models (ACM) to generate a curve that closely fits the skull border and introduce a novel algorithm to automatically model the implant. However, this method presents the same limitations as surface interpolation-based methods, due to the lack of information related to the inner part of the defect area. Indeed, when dealing with large defects, the information provided by the contours is usually not sufficient to properly reconstruct the missing region.

Recently, methods based on deep learning have been proposed. There are two main approaches in this space: the first pertains reconstructing the entire skull and then extracting the defect by subtraction^6–8^; the second approach consists in directly predicting the implant^15–17^. Most of the solutions have been tested to aid the design of implants for cranioplasty, i.e. all the defects are located in the neurocranium. Moreover, the neural networks used are either 2D CNNs that complete one slice at the time or 3D CNNs that work on a voxelized version of the skull. The former are efficient but their reconstruction may lack global coherence, while the latter require a fine quantization to be able to reconstruct the detail with an appropriate level of resolution, which results in large voxels grids (where about 90% of the grid is empty^17^). Among papers working on slices, Li et al.^18^ proposed a two-step reconstruction that exploits two neural networks. The first network aims at reconstructing a low-resolution version of the skull, while the second is trained to increase the level of detail starting from the low-resolution model. Other solutions^19,20^ exploit an autoencoder architecture and a 3D U-Net^21^, respectively, to predict the region to be reconstructed. As for 3D CNNs, Wu et al.,^15^ proposed a autoencoder with skip-connections and 3D convolutions, which starts from the defective skull model and generates the complete skull. Kodym et al.^6^ introduced an open dataset and proposed a framework for fully-automatic craniofacial skeletal reconstruction. Successively, they proposed a multi-branch reconstruction model^7^ which is trained on two different types of data: the ground truth data corresponding to the skull region to be reconstructed; and the expert-designed cranial implant shape.

To overcome the limitations of previous approaches, we investigate a different and under-explored path, which relies on leveraging efficient data structures such as 3D point clouds. Moreover, differently from all previous work, we tackle reconstruction also in the splanchnocranium.

Only one recent work^22^ partially addresses the splanchnocranium area (reconstructing midfacial bones) by exploiting Generative Adversarial Networks (GANs) to reconstruct realistic slices after artificial defects have been injected. However, the artificial defects add simple geometric volumes such as spheres and cylinders to the midfacial bone area instead of simulating resection of a malformation as we do in our study. Contemporary work investigated the use of point clouds for cranioplasty^23^. They employ a Generative Adversarial Network (GAN) with an encoder-decoder architecture for the generative task and a fully connected network for the discriminator. The encoder is a modified version of PointNet^24^ that generates a shape code which is then decoded through an MLP with one hidden layer to obtain the completed point cloud. Compared to our work, they address only cranioplasty, do not model the problem as shape completion, work with limited resolution point clouds up to 1024 points, and do not reconstruct the skull as a mesh.

## Methods

### Ethical approval

The majority of the experiments in this work were conducted on the CQ500 dataset^5^, which is publicly available for research. For the retrospective study of the clinical case, informed consent was obtained for the utilization of the CT image of the patient, with explicit clarification that the patient had undergone prior treatment before the simulation, and that this did not influence the treatment in any manner. This study was carried out in accordance with the Declaration of Helsinki.

### Dataset

**Figure 1:**
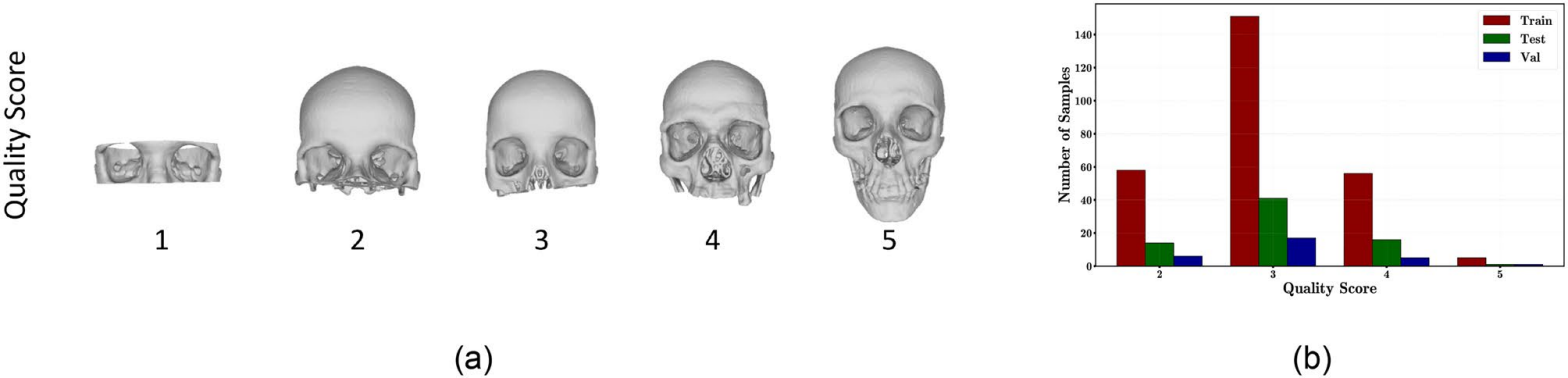
(**a**) Examples for different Quality Score, (**b**) train, validation and test splits obtained considering the same proportion of samples w.r.t the quality scores.

In this work we focus on the CQ500 dataset^5^, which contains 403 anonymized CT scans in Digital Imaging and COmmunications in Medicine (DICOM) format. Since these scans were acquired for a variety of purposes, most of the skulls are partial. Hence, we asked an expert to label each scan with a *Quality Score* metadata, which provides a comprehensive assessment of the quality of the skull scan that considers the extension/completeness of the shape. It can assume a value between 1 and 5; the higher the value, the better the overall quality, as shown in Fig. 1a.

The skulls that present a severe deformity or damage (e.g. CT scans of non-eumorphic patients, CTs showing fractures and CTs the slice thickness of which did not allow proper 3D reconstruction upon segmentation) have been discarded. In addition, we decided to discard the skulls with quality score 1 because the observed skull region was too limited. Therefore, the final dataset contains 385 skulls in total and each sample has between 250,000 and 400,000 vertices. The dataset is split into train (270), validation (29) and test (71) sets. The proportion of samples for each split w.r.t the quality score is shown in Fig. 1b.

#### Pre-processing

In order to obtain the 3D model from the DICOM format file, the CT scans are processed using the Materialise’s Interactive Medical Image Control System (MIMICS)^25^ software. Each slice is then filtered so as to derive only the bone structure (high density) of the head. Finally, all the vertebrae are removed to obtain the final mesh. The meshes are moved to a Natural Head Position (NHP), i.e., a standardized and reproducible position of the head in an upright posture. Successively, each mesh is aligned with respect to a reference skull according to 3 points, two on the frontozygomatic sutures and one on the basion. This initial alignment is further refined by exploiting the Iterative Closest Point (ICP) algorithm^26^.

**Figure 2:**
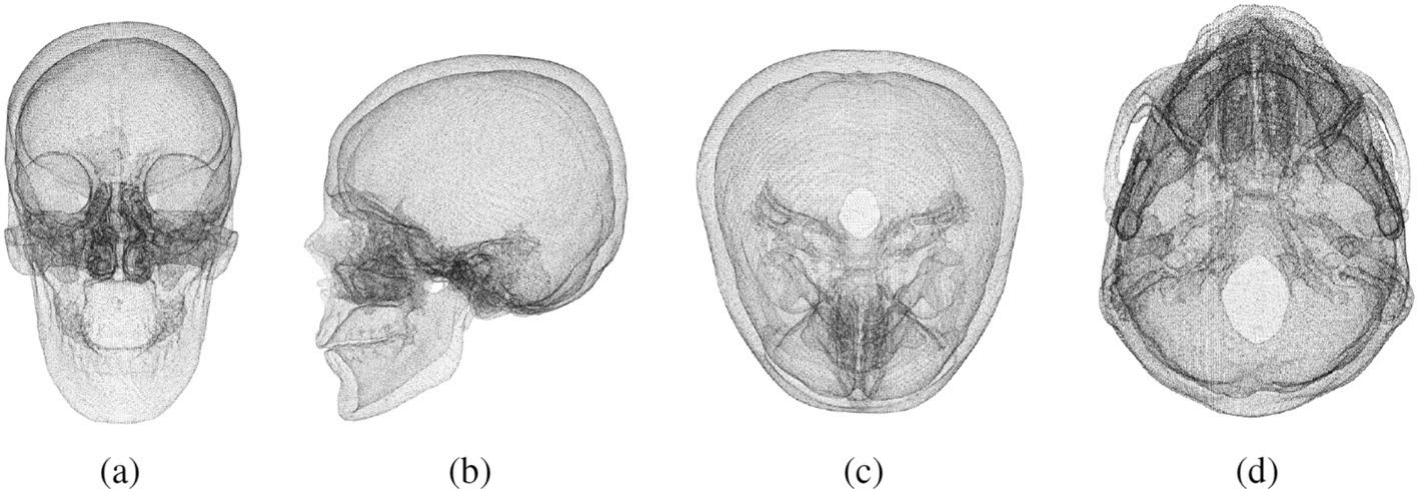
Due to the internal points, two main volumes can be distinguished. The innermost is made of points that can be removed to simplify the point clouds since the most important outcome of the reconstruction process is the shape of the external surface. (**a**) Frontal view, (**b**) Left Lateral view, (**c**) Parietal view, (**d**) Basilar view.

Since we address the defect reconstruction problem as a point cloud completion task, we consider only the vertices of the meshes in our dataset. However, these meshes have a very large number of internal vertices (Fig. 2), which are mostly related to the internal skull bones structures. Reconstructing these points has limited clinical relevance since the most important outcome of the reconstruction process is the shape of the external surface^7^. To remove the internal vertices, we designed the following pipeline:

1. Take snapshots of the 3D mesh from different angles.
2. Extract the depth map from each snapshot.
3. Convert the depth information into point clouds.
4. Merge all the point clouds.
5. Simplify the final point cloud.

**Figure 3:**
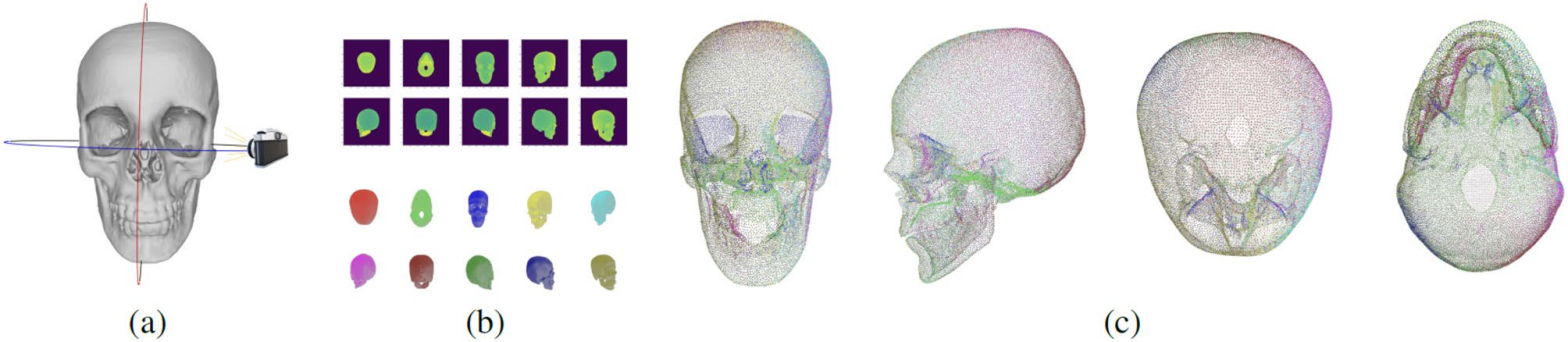
Internal points removal pipeline: **a** Snapshots of the 3D model from different angles, (b-top) Depth maps of the snapshots, (b-bottom) Point clouds obtained from depth information, **c** Final result.

The first step consists in acquiring snapshots of the skull mesh with virtual cameras from different points of view, Fig. 3a.

In order to capture the overall external structure of the skull, eight snapshots are taken, rotating the camera around the vertical axis in steps of 45 degrees. Two additional views are acquired, one parietal and one basilar. From each of these snapshots, the depth map is extracted (Fig. 3b) and converted into a point cloud by simply projecting back the depth image into the 3D points given the camera rotation and translation of a specific snapshot, so as to keep all the point clouds in the same reference system (Fig. 3c). The point clouds from every camera position are concatenated together to obtain the final point cloud of the skull without the internal points. This cloud contains about 100–300k points, while shape completion networks usually work on clouds with 2048 points. Therefore, the point cloud is further simplified through the Poisson Disk Sampling (PDS)^27^ to 40k points. The final result is shown in Fig. 3c.

**Figure 4:**
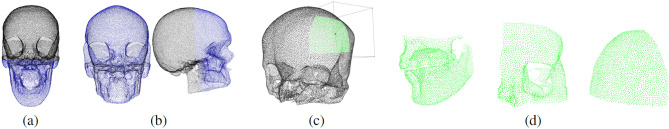
Defect Injection: (**a**, **b**) in blue, points in the area where a random point is selected for point clouds with quality score 5 (**a**) and 4 (**b**); (**c**) defect creation: the random point used as the center of the cuboid with square base is shown in red, while the selected points that will be removed to create the defect are in green; (**d**) some defects.

### Defect injection

Defects were generated artificially since the dataset only comprises normal skulls. The artificial defects were created by removing skull portions from the complete point clouds. Figure 4 shows how a defect is created. A random point is selected as the center of the defect. For skulls whose quality score is 4 a random point on the region of the splanchnocranium is taken, Fig. 4b, while for skulls of quality score 5, a random point on the maxilla and mandible part is taken, Fig. 4a. We select points in these specific regions of the more complete CT scans to balance the number of defects across skull regions. Complete CT scans, such as those having quality score 4 and 5, are likely to be acquired when working on defects affecting the splanchnocranium region. The chosen point is then used as the center of a rectangular cuboid with a square base, having random height and base side between 3cm and 10cm, as shown in Fig. 4c. Every point that falls into the cuboid is removed from the point cloud. It is worth highlighting that the partial clouds for the train set are created online during training, so they are always different, while the validation and test sets have been created off-line and kept fixed.

### Normalization

**Figure 5:**
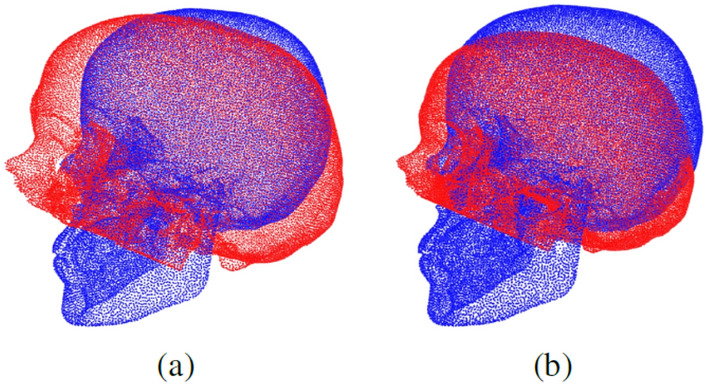
(**a**) Alignment after ShapeNet- like normalization. (**b**) Alignment after using a fixed scale factor *m* for normalization.

Each point cloud $P = \{ p_{i} \} ^{N}_{i = 1}$ is composed by *N* points, $p_{i} = (x_{i}, y_{i}, z_{i})$, and is normalized to guarantee that each coordinate takes values in $\{-1, 1\}$. Point clouds in datasets like ShapeNet^28^ are normalized by subtracting their centroid, to center them in the origin (0, 0, 0), and by dividing them with a scalar value which is obtained as $m =$ $\max _{p_{i} \in P} ( \sqrt{\sum (x_{i}^{2} + y_{i}^{2} + z_{i}^{2})} )$. For our dataset, this type of normalization is detrimental, because there are both complete skulls, with a quality score of 5 and partial skulls, for example the skulls without the jaw. Therefore, depending on the completeness of the skull and the dimension of the defect, *m* may assume very different values, which in turn would cause changes in the skull scale across different quality scores, as shown in Fig. 5a. Since input normalization is mainly used to ease optimization of neural networks, it should not create spurious large displacements between corresponding anatomical structures in different samples. To overcome this issue, we select $m = 155 mm$ which is the maximum scale of the point clouds in the training set and use this value as a fixed scale factor for all skulls. In this way, the partial point clouds are normalized by subtracting their centroid and then diving by the constant *m*. The result is shown in Fig. 5b. Both the partial and the complete clouds are centered with respect to the centroid of the former in order to keep them aligned. Note that we cannot simply normalize the complete point clouds before creating the defects because, in a real deployment scenario, we may not have the original complete skull.

### Methodology

**Figure 6:**
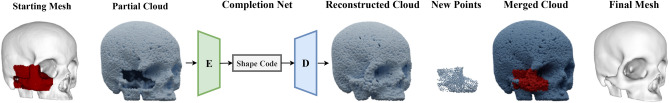
Complete pipeline for the proposed skull reconstruction framework.

Our system is meant to be used to provide a plausible completion of a skull with a missing part to help the clinician plan surgery or design the implant. Hence, we assume that the mesh has been extracted from CT scans by means of standard techniques like marching cubes^29^. Then, given a defective region selected by the surgeon (area in red in the leftmost subfigure of Fig. 6), we perform the steps outlined in Figure 6. We first sample a partial point cloud not considering points from the selected region. The core step of our pipeline is to cast the task as a shape completion task. In shape completion, a partial point cloud $P = \{ p_{i} \} ^{K}_{i = 1}$ is provided as input to a neural network, which will process it and provide as output a complete point cloud $\hat{P} = \{ p_{i} \} ^{N}_{i = 1}$, with $N > K$, with the missing part reconstructed. Hence, the partial point cloud is completed by one of the tested point cloud completion networks, and only the points inside the defective region are retained and concatenated with the partial input point cloud. Finally, a post-processing procedure generates the final mesh. Since the merged point cloud holds the normal vectors only for the partial input point cloud, which were inherited from the input mesh, a normal estimation algorithm is applied in order to estimate the normal vectors for the reconstructed region. In particular, the direction of the normal vector for a vertex is computed via Principal Component Analysis and it corresponds to the direction of the eigenvector associated with the smallest eigenvalue of the local covariance matrix obtained by taking into consideration the $k_c$ nearest neighbors of that vertex. Then, by applying the consistent tangent plane algorithm^30^, normals are consistently oriented by propagating their sign along a graph built on top of the point cloud by connecting each point with its $k_{tg}$ nearest neighbors. Finally, the Poisson reconstruction algorithm^31^ is run to derive the triangular mesh. We note that our aim is not to provide a better method for mesh extraction from CT scans, and we used the standard marching cube algorithm when we needed to perform such a step in our pipeline.

We modified and tested the following state-of-the-art neural networks for shape-completion: FoldingNet^32^, PCNet^33^, PoinTr^34^, PointAttN^35^, SnowflakeNet^36^, PMP-Net++^37^ and VRCNet^38^. Each proposal relies on different strategies and specific layers to perform the completion but in general, they follow an encoder-decoder architecture. In particular, the encoder starts from the partial point cloud and generates a compact latent code that summarizes the information about the input shape; the decoder, starting from this latent code and the partial input, reconstructs the complete shape.

Architectures that exploit a folding-based decoder^32,33^ assume that a 3D shape can be obtained from the deformation of a 2D grid (folding operation). Instead of performing the folding operation locally, FoldingNet^32^ applies two consecutive folding procedures over a fixed-size grid in order to generate the whole object shape. Differently, PCNet^33^, proposes a coarse-to-fine generation process. In the first phase, a skeleton of the shape is obtained, then a patch folding operation centered on each point of the coarse representation is performed so as to obtain an high-resolution point cloud.

Transformer-based networks^34–36^ exploit the attention mechanism to perform shape completion. With the geometry-aware transformer block, PoinTr^34^ introduced a transformer encoder-decoder architecture that addresses shape completion as a set-to-set translation task. It transforms the partial point cloud into a series of point proxies (per-point features) and then generates a set of point proxies, for the missing part, that are transformed back into 3D point coordinates with a folding operation. SnowflakeNet^36^ utilizes the transformer structure in the decoding phase. In particular, the authors introduced the Snowflake Point Deconvolution (SPD) which generates point displacements in a parent-child fashion, akin to the growth of a snowflake. Each SPD uses information from the previous splitting, which makes it possible to predict detailed local geometries. Differently, PointAttN^35^ exploits only self-attention and cross-attention mechanisms to process point clouds in a per-point manner through two novel layers: Geometric Details Perception and Self-Feature Augment.

Differently from the approaches described so far, PMP-Net++^37^ does not generate points, instead, it predicts a unique Point Moving Path which moves each point of the partial input to obtain an approximation of the complete shape. At inference time the network is fed with several downsampled versions of the partial input and the final high-resolution point cloud is obtained by concatenation. Finally, VRCNet^38^ consists of two subnetworks: the probabilistic Modeling Network (PMNet) and the Relational Enhancement Network (RENet). The first subnetwork, PMNet, predicts a coarse point cloud. It uses a variational autoencoder to align the distribution of the complete and partial clouds in the learned latent space. RENet, instead, learns effective multi-scale local point features thanks to a specialized layer design.

**Table 1:** Hyperparameter settings for the considered shape completion networks.

Network	Num. input points	BS	Epochs	LR	Optimizer	Scheduling
FoldingNet	35000	8	300	0.0001	Adam	None
PCNet	35000	16	300	0.0001	Adam	$\gamma = 0.7$ every 80 e
VRCNet	15000	4	300	0.0001	Adam	$\gamma = 0.7$ every 80 e
PoinTr	15000	8	600	0.0005	AdamW	$\gamma = 0.9$ every 21 e
PointAttn	8192	4	400	0.0001	Adam	$\gamma = 0.7$ every 40 e
PMP-Net++	10000	16	400	0.001	Adam	$\gamma = 0.5$ every 100 e
SnowflakeNet	35000	16	800	0.001	Adam	$\gamma = 0.5$ every 100 e

## Results

In this section we report on the performance obtained by the considered shape completion networks on the skull defect reconstruction task. We first outline the metrics adopted in our experiments, then we provide some details about the hyperparameter settings adopted for training the different models. Finally we show both quantitative and qualitative results.

### Evaluation metrics

All metrics are computed after the de-normalization of the point clouds and considering only the reconstructed region, not the entire shape. In particular, we compute Accuracy, Completeness, the Chamfer Distance, the Earth Mover Distance and the F-score between the ground-truth points of the defect and the reconstructed region.

*Accuracy* Given two point clouds $P_{GT}, P_{Rec}$ the Accuracy is computed as follows:

1 $$\begin{aligned} \frac{1}{|P_{GT}|} \sum _{x \in P_{GT}} \min _{y \in P_{Rec}} \Vert x - y \Vert _{2} \end{aligned}$$

i.e., for each point in the ground-truth, its nearest neighbor in the reconstruction is found and their distance is computed. It estimates how close the output’s points are to the ground truth.

*Completeness* Given two point clouds $P_{GT}, P_{Rec}$ the Completeness is computed as follows:

2 $$\begin{aligned} \frac{1}{|P_{Rec}|} \sum _{y \in P_{Rec}} \min _{x \in P_{GT}} \Vert x - y \Vert _{2} \end{aligned}$$

i.e., for each point in the reconstructed point cloud its nearest neighbor in the ground truth cloud is found and their distance is computed. It measures how well the ground truth is covered by the output point cloud.

*Chamfer distance (CD)* Given two point clouds $P_{GT}, P_{Rec}$ the Chamfer distance is computed as follows:

3 $$\begin{aligned} \frac{1}{|P_{GT}|} \sum _{x \in P_{GT}} \min _{y \in P_{Rec}} \Vert x - y \Vert _{2} + \frac{1}{|P_{Rec}|} \sum _{y \in P_{Rec}} \min _{x \in P_{GT}} \Vert x - y \Vert _{2} \end{aligned}$$

i.e., the sum of Accuracy and Completeness. It provides an overall measure of quality of the completion.

*Earth Mover Distance*^39^ *(EMD)* Given two point clouds of the same size *P* and *Q* the EMD is computed as follows:

4 $$\begin{aligned} \min _{\Phi : P \rightarrow Q} \frac{1}{| P |} \sum _{x \in P} \Vert x - \Phi (x) \Vert _{2} \end{aligned}$$

With respect to the Chamfer distance, the Earth Mover’s distance can be computed only if the two point clouds have the same number of points, since it measures the average distance between corresponding points in an optimal one-to-one matching. Thus, in order to compute the EMD, we downsampled the point cloud with the highest number of points.

*Precision, Recall and F-Score* are computed as function of a distance threshold $\tau$. F-Score^40^ is defined as the harmonic average of precision and recall. If $P_{GT}$ is the ground truth point cloud and $P_{Rec}$ is the output of the network, precision and recall are computed as follows:

5 $$\begin{aligned} P(\tau ) =\frac{1}{|P_{Rec}|} \sum _{r \in P_{Rec}} [ \min _{g \in P_{GT}} \Vert g - r \Vert<\tau ], \ \ \ R(\tau ) = \frac{1}{|P_{GT}|} \sum _{g \in P_{GT}} [ \min _{r \in P_{Rec}} \Vert g - r \Vert <\tau ], \ \ \ \text {F-Score }(\tau ) = \frac{2P(\tau )R(\tau )}{ P(\tau ) + R(\tau ) } \end{aligned}$$

**Table 2:** Metrics computed over the missing region with respect to the ground truth.

	Accuracy[mm]		Completeness[mm]		CD[mm]		EMD[mm]		F-Score(3mm)	
	*All*	*QS5*	*All*	*QS5*	*All*	*QS5*	*All*	*QS5*	*All*	*QS5*
FoldingNet	3.7306	3.5601	5.579	7.9385	9.3096	11.4986	2.9927	3.3926	0.4158	0.3108
PCNet	2.7411	2.6634	2.4921	3.0371	5.2332	5.7005	3.4867	3.9887	0.7071	0.6681
VRCNet	2.7092	2.8547	2.671	3.3191	5.3803	6.1738	3.3725	3.9003	0.6885	0.6094
PoinTr	1.9019	2.3541	2.8119	3.3918	4.7138	5.7459	2.8186	3.0383	0.7659	0.6147
PMP-Net++	1.8104	2.048	2.6404	3.0851	4.4508	5.133	2.5617	2.4918	0.7475	0.6497
PointAttN	**1.4468**	**1.842**	1.8262	2.1443	**3.2731**	**3.9863**	2.2857	2.4039	**0.8929**	**0.8292**
SnowflakeNet	1.6147	2.0203	**1.6983**	**2.0949**	3.313	4.1152	**2.0461**	**2.2925**	0.8895	0.8178

In bold the best result, underlined the second best result.

### Training settings

Table 1 provides an overview of the hyperparameters employed for each model. In particular, we used the hyperparameters that provided the best results in the original works. For all our experiments we employed the official implementations provided by the authors. Training and validation have been performed with a NVIDIA GeForce RTX 3090 Ti with 24GB of memory.

### Quantitative results

**Figure 7:**
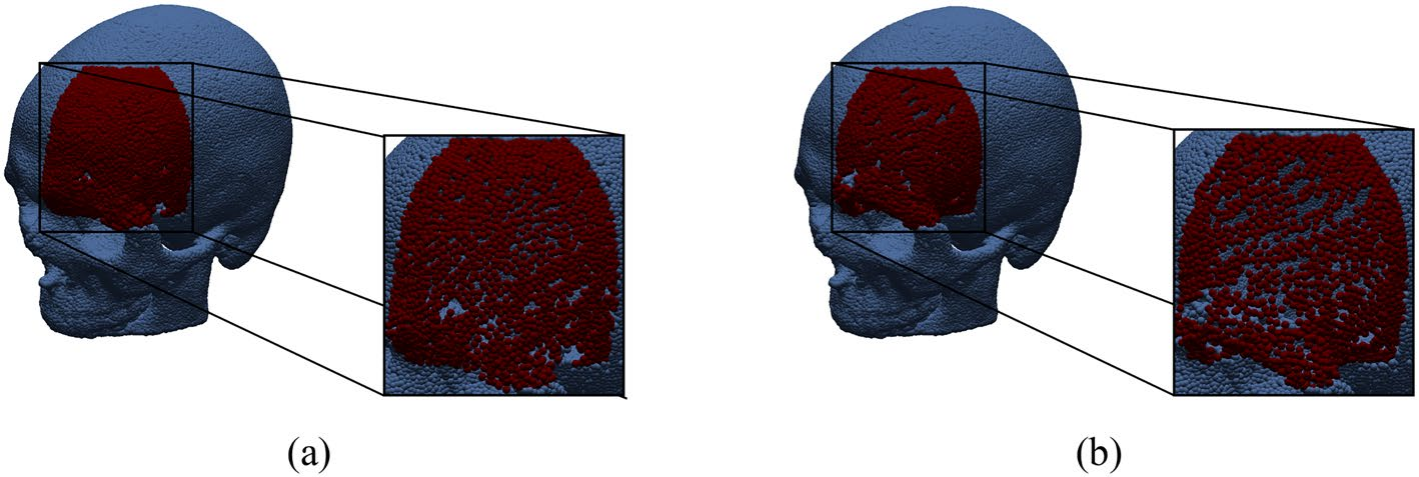
(**a**) Reconstruction obtained from SnowflakeNet, (**b**) reconstruction obtained from PointAttN.

**Figure 8:**
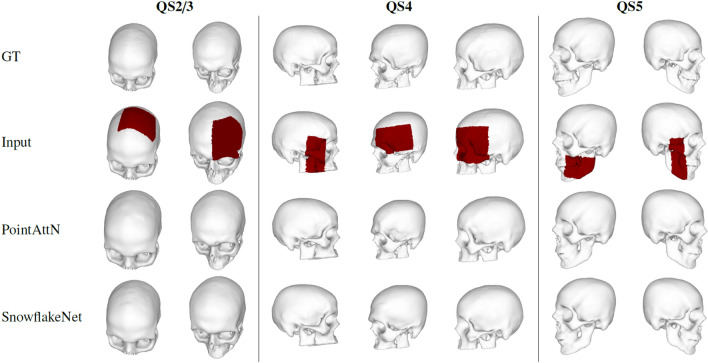
Qualitative reconstruction results.

Table 2 reports the metrics computed on the test set, considering only points in the defect. We report results averaged across all skulls in the test set as well as on skulls with quality score 5, so as to highlight the performance in reconstruction of the facial skull and unlike previous work focused on reconstruction of the neurocranium^6–8^. The networks with the best results are SnowflakeNet and PointAttN. They can complete the missing region with both high accuracy and completeness and this reflects positively on the normal estimation and the surface reconstruction processes, as shown in the qualitative results (see Figure 8). It is worth noticing that the completeness of SnowflakeNet is lower than PointAttN. This is due to the fact that the former shows in general a more uniform point distribution in the reconstructed region as shown in Figure 7.

It can also be observed how the reconstruction results get worse if we only consider skulls with quality score 5. This is mainly motivated by two reasons: (i) the completion task is far more difficult because this area of the skull has a very complicated geometry; (ii) only 5 skulls with the mandible were available in the training set. Nonetheless, the networks were able to generalize sufficiently well to unseen data also in these challenging cases, i.e. the average CD distance is about 4 mm when considering only skulls with quality score 5. On QS5 skulls, PointAttN seems the most effective shape completion network: it produces the best results for all metrics but completeness, where however the gap with SnowFlakeNet reduces significantly with respect to the metric dealing with the whole dataset.

### Qualitative results

In this section, we show some qualitative reconstruction results obtained with the two best-performing networks, namely SnoflakeNet and PointAttN. The leftmost part of Fig. 8 shows the final meshes obtained for skulls with a quality score of 2 and 3 of the test set. When the artificial defect is on the frontal or parietal bone of the skull, the reconstructed patch matches perfectly the partial skull with a smooth surface. When the defect is localized on the cranium region, the reconstruction is performed only on the external surface of the skull, which is the most relevant part to then design an implant. The central part in Fig. 8 shows some samples with the defect on the zygomatic area of the cranium, for a skull with a quality score of 4. Even if the topology of this region is more complex, the networks can capture the local details and reconstruct the missing region properly. Finally, the rightmost part of Fig. 8 shows some reconstruction results for the jaw region for skulls with a quality score of 5. This area presents fine-grained details that are accurately reconstructed.

**Figure 9:**
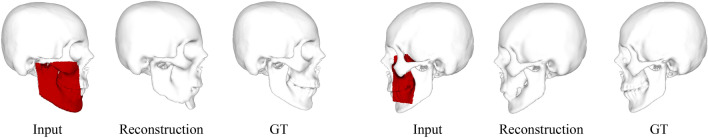
Examples of failed reconstructions.

Some failure cases are shown in Fig. 9. They can be either due to a wrong reconstruction of the shape, which creates a non-realistic skull, as shown in the example in the first row, or poor normal estimation in the presence of a reasonable shape in the point cloud, shown in the second row, which leads to holes in the final mesh.

### Reconstruction of the orbit subunit

In this section, we report the results of experiments aimed at assessing if a model trained to reconstruct only specific subunits of the skull may outperform the holistic approach presented so far. Driven by this motivation, the defect injection pipeline has been modified to generate defects in the orbit area of the skull. According to this defect generation pipeline, new train and test sets have been produced with the same augmentation strategy to balance the dataset in terms of quality score. The PointAttN network has been trained from scratch and a specialized model focusing on orbit defects has been obtained. The new Orbit-Specific network has been compared with the results obtained from the Full-Skull model, i.e. the network trained with the defect injection already described, while both networks have been evaluated on the new test set. In Table 3 quantitative results are reported.

**Table 3:** Metrics computed over the missing region in the reconstruction of the orbit subunit.

	Accuracy (mm)	Completeness (mm)	CD (mm)	EMD (mm)	F-Score (3mm)
Full-Skull	1.8045	2.1286	3.9331	2.5634	0.8302
Orbit-Specific	1.8528	2.0591	3.912	2.54915	0.8313

The results for both networks are similar, this suggests that the neural network has enough capacity to learn to repair defects in all areas, and for this reason, there are no particular benefits in training a specialized model.

## A clinical use case

**Figure 10:**
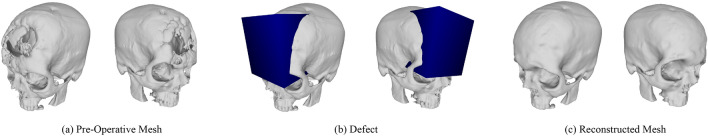
Example of a clinical case reconstruction.

We applied the methodology to the cranium of a patient previously treated for a secondary revision of an extensive fronto-orbito-ethmoidal fracture with loss of bone (Fig. 10a). The patient had been treated via the application of a customized implant designed according to a non-defective cranial template, slice-based reconstruction and freehand modelling. This was necessary due to the median position of the defect, its size and the complex topology of the fronto-orbito-ethmoidal region involved, especially due to the compound curvature of the bone surface in the fronto-nasal region. The affected area was bound by the smallest possible bounding box with sides parallel to the reference axes, according to the training pipeline, resulting in the exclusion of the medial half of the upper orbital rim and the Nasion region (Fig. 10b). The resulting reconstruction (Fig. 10c) was valued by expert surgeons as qualitatively compatible with the native patient’s anatomy with excellent rendition of the compound curved surfaces of the involved region. This validates the claim that the proposed methodology can provide a patient-specific guide surface for implant design which could cut labor-intensive and operator-dependent tasks.

## Conclusions

In this work, we have investigated the feasibility and effectiveness of performing skull reconstruction, routinely performed by clinicians when planning a surgery, through shape completion. To the best of our knowledge, our work delineates the first solution that exploits the advances of deep learning for point cloud completion in order to create an automatic clinical aid for maxillofacial surgical planning. We also propose a new dataset enriched with virtual resections, suitable to study the performance of shape completion models on this challenging task. Our dataset allows researchers to investigate on surgical planning in both in the neurocranium and splanchnocranium regions. We have presented a full pipeline that automatizes the creation of meshes of eumorphic skulls given as input the resection volume and the incomplete shape. Experimental results on a large pool of state-of-the-art shape completion networks have identified the best-performing models and shown the feasibility and effectiveness of the proposed approach to maxillofacial surgical planning. Even if our pipeline already provides promising results, the decoupled processes of point cloud reconstruction and normal estimation for surface reconstruction are inefficient and do not allow the model to reason jointly on point positions and normal orientations. For this reason, exploring solutions that may predict also the normals holds the potential to further improve results. In addition, the implant extraction process requires prior knowledge of the size and position of the missing region of the input. Hence, to fully automate the process, future work will explore methods that directly predict the missing region of the input partial cloud. Moreover, collecting a dataset of real resections and testing our approach on them is a natural follow-up of our research. Finally, an interesting research direction could be to extend our pipeline to handle not only missing geometry, but also displaced anatomy, to perform automatic planning of reduction of fractured fragments. A first way to tackle this problem while leveraging the results of the current study could be to remove the displaced anatomy, complete the skull with the proposed pipeline, and then register the displaced anatomy to the proposal of the network, to estimate a tentative roto-translation for each part. We plan to explore such direction in future work.

## Data Availability

The CQ500 dataset (http://headctstudy.qure.ai/dataset) is available upon request to qure.ai. Only the CT scan used to test the algorithm in the clinical use case relied on in-house data. The scan cannot be shared.
